# Value of the cuff leak test is limited

**DOI:** 10.1186/s13054-015-1152-x

**Published:** 2015-12-24

**Authors:** Wei Wang, Yu Zhou, Hua-sheng Tong, Lei Su, Ling Zhao

**Affiliations:** Department of Critical Care Medicine, Guangzhou General Hospital of Guangzhou Military Command, Southern Medical University, Guangzhou, Guangdong China; Department of Critical Care Medicine, Zhuhai Hospital of Jinan University, Zhuhai People’s Hospital, 79 Kang-ning road, Zhuhai, 519000 China

We read with interest the recent *Critical Care* review of post-extubation laryngeal edema and stridor resulting in respiratory failure [1]. We mostly agree with the authors’ opinion in that article except that the cuff leak test (CLT) was proposed as a standard extubation algorithm. We have two reasons to disagree.

First, although the CLT has been widely used for the prediction of post-extubation laryngeal edema, evidence for the predictive value of the CLT is conflicting. Most studies on the CLT document a high specificity and a low sensitivity, and this means that patients with a negative test have a low probability of developing post-extubation stridor (PES) but that patients with a positive CLT may not develop PES. In a recent systematic review, Ochoa et al. evaluated the accuracy of the CLT for reintubation secondary to upper airway obstruction; the sensitivity was 0.63 (95 % confidence interval (CI) 0.38–0.84), and the specificity was 0.86 (95 % CI 0.81–0.90) [2]. Shin et al. report that the CLT does not reliably identify those patients who will require reintubation in a trauma population [3]. Similarly, a recent study by Patel et al. demonstrates that the CLT or a combination with laryngeal parameters failed to accurately predict PES [4].

Second, the CLT is regarded as a simple and non-invasive procedure, but that does not mean this procedure is totally safe for patients. Ventilator-associated pneumonia (VAP) is one of the most frequent hospital-acquired infections and is associated with higher mortality, morbidity, and costs. Contaminated secretions that spill over the endotracheal tube cuff and leak down to the lungs are considered a significant pathogenic mechanism of VAP [5]. Although the related evidence is lacking, it is reasonable to posit that the CLT should increase the risk of oropharyngeal and subglottic secretions into the airway during cuff deflation and mechanical ventilation.

The poor predictive accuracy suggests that the CLT is an unstable clinical prognosticator, and we consider that the CLT should be applied for patients at high risk and that the excessive use of CLT is inappropriate.

## Abbreviations

CI: Confidence interval
CLT: Cuff leak test
PES: Post-extubation stridor
VAP: Ventilator-associated pneumonia
